# Genome-wide identification, characterization and phylogenetic analysis of Dicer-like (DCL) gene family in Coffea arabica

**DOI:** 10.6026/97320630015824

**Published:** 2019-12-11

**Authors:** Md Parvez Mosharaf, Zobaer Akond, Md Hadiul Kabir, Md Nurul Haque Mollah

**Affiliations:** 1 Bioinformatics Laboratory, Department of Statistics, University of Rajshahi, Rajshahi-6205, Bangladesh

**Keywords:** Dicer-Like, in silico approach, Coffea arabica

## Abstract

A fine-tuned RNA interference (RNAi) pathway has been developed by plants to restrain distinct biological processes in various life stages including stress responses, development and maintenance of genome integrity. The Dicer-Like (DCL) proteins starts the RNAi process by producing complementary double-stranded RNAs (dsRNAs) into small RNA duplexes (21-24 nucleotides) trigger the RNAi process. Nevertheless, these members of RNAi pathway have not been deciphered in one of the most economically important plant coffee (*Coffea arabica*). Therefore, it is of interest to report the identification and phylogenetic analysis of the DCL genes in C. arabica. We report 5 DCL genes and categorized them into three significant groups to interpret the evolutionary relationship with DCLs of the model plant *Arabidopsis thaliana*. Moreover, the subcellular location of the reported DCL proteins and the associated cis-acting regulatory elements were also identified and discussed in this report. The cis-regulatory elements indicated the biological and molecular functional diversity of the identified DCL genes related with plant growth and development. The present findings will provide a better basis for further experimental research on RNAi pathway genes in C. arabica.

## Background

Plants have developed a fine-tuned RNA interference (RNAi) pathway to regulate various biological processes, including development, stress responses and maintenance of genome integrity. In plants most of the biological and molecular functions are triggered by two types of small RNA called microRNA (miRNA) and short interfering RNA (siRNA) [1,2]. These small RNA contain 21-24 nucleotides, which are actually involved with both transcriptional and post-transcriptional RNA mediated gene silencing [3]. The mechanism of RNAi pathway depends on the direct involvements of the Dicer-like (DCL), Argonaute (AGO) and RNA-dependent RNA polymerase (RDR) genes. The main element of RNAi mechanism is the processed mature siRNAs, which are produced from the double-stranded RNAs [4,[5]. These siRNA biogenesis process fully introduced by one of the inevitable member of RNAi called DCL proteins having the functional domains, named DEAD/ResIII, Helicase_C, Dicer_Dimer, PAZ, RNase III and DSRM [6]. TheDCL proteins specifically process complementary double-stranded RNAs (dsRNAs) into small (21–24 nucleotide) RNA duplexes which siRNAs actually initiate the whole RNA interference process. These siRNA degrade the target homologous RNAs with the complementary sequence to these siRNAs with the help of RNA-induced silencing complex (RISC) and AGO proteins [7]. In case of plants, the DCL, AGO and RDR gene families contain multiple genes belonging to distinct RNAi pathways [8,[9]. These RNAi genes have been characterized in many species with their functional activities, which helps to make genetic development for those plants. These genes were identified and characterized into Arabidopsis thaliana (http://www.arabidopsis.org/), rice [10], cucumber [11], maize [12], tomato [13], tobacco [14], foxtail mille t[15], and grapevine [16] and so on. Moreover, in silico identification of RNAi genes in many species had been conducted before [17].

These efficient and indispensable components of the RNAi pathway has not been identified and characterized into one of the most economically important plant, coffee (C. arabica), which provides more than 60% coffee of the total coffee production all over the world according to the report from United States Department of Agriculture [18]. In this study, we have taken first initiative to detect the inevitable DCL gene family members into this commercially important plant. Here we provided a comprehensive genome wide in silico analysis to identify, characterize and check the phylogenetic evolutionary relationship of coffee DCL genes including functional domain conservation, gene structure, genomic localization and other physicochemical propertiescompared with the DCL from model plant Arabidopsis thaliana. In addition, the subcellular location of the reported DCL proteins and the cis-acting regulatory elements associated with the C. arabica DCL genes were also retrieved in this study.

## Methodology

### Identification of DCL Genes:

**Figure 1:**
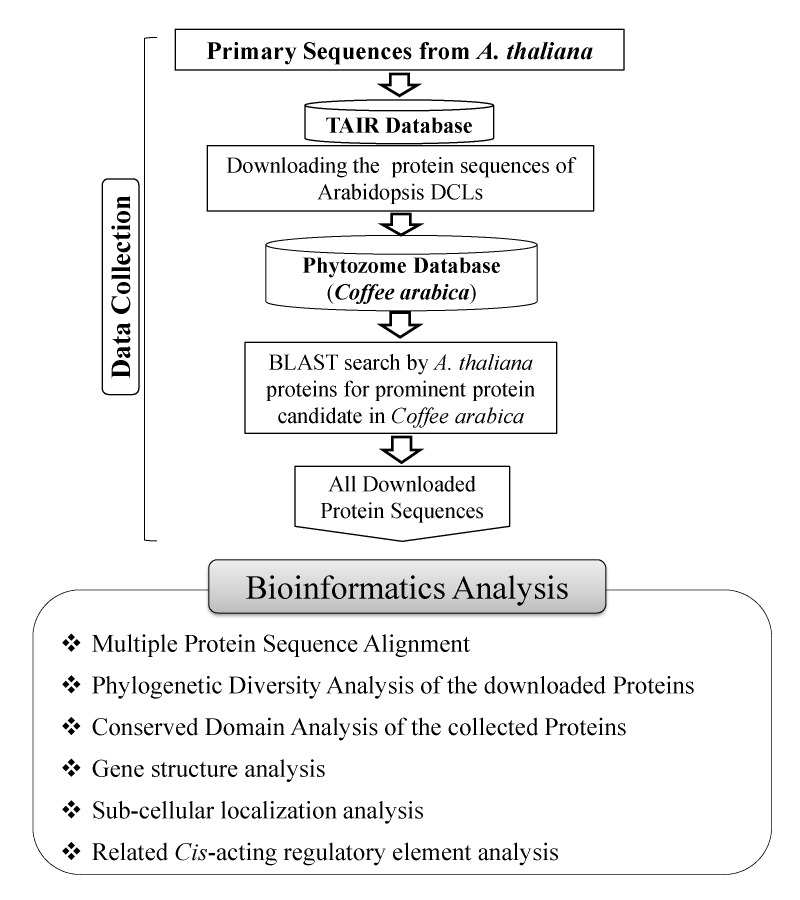
The global working flowchart of this study

For this genome-wide investigation, the probable DCL protein sequences were collected from the well-known plant full genome database Phytozome (https://phytozome.jgi.doe.gov/pz/portal.html). To identify DCL protein sequences in Coffee (Coffea arabica), the Arabidopsis DCL proteins sequences were used as query to search by the Basic Local Alignment Search Tool (BLASTP) program against C. arabica genome into the Phytozome database. We primarily downloaded the paralogsprotein sequences synthesized from only the primary transcripts of C. arabica considering the score (≥50) and E-values. Along with these, the related genomic information was also recorded. The newly identified DCL genes of C. Arabica are named based on nomenclature with the help of phylogenetic relationship study. The closeness of the DCL proteins from C. arabica in the phylogenetic tree to the related DCL proteins of A. thaliana was the naming criterion of the newly identified RNAi proteins. The entire working flowchart is given in (Figure 1).

### Sequence alignment and phylogenetic analysis

The multiple sequence alignments of the encoded probable CaDCL protein sequences were utilized through the Clustal-W method [19] in the MEGA5 program [20]. To construct the phylogenetic evolutionary relationship among the aligned protein, the Neighbour-joining method [21] with the 1,000 bootstrap replicates [22] were used. The Equal Input method [23] calculated the evolutionary distances.

### Conserved domain identification:

The NCBI-CD database (http://www.ncbi.nlm.nih.gov/ Structure/cdd/wrpsb.cgi) and the Pfam (http://pfam.sanger.ac.uk/) were utilized to investigate the conserved domains of all retrieved sequences. The conserved domain part analysis helped to make the shortlist of the best candidate to be the CaDCL member in C. arabica genome. The proposed genes confirmed to have the DEAD/ResIII, Helicase_C, Dicer_Dimer, PAZ, RNase III and DSRM domains, which are the core component of the CaDCL proteins, revealed by the other studies before [10-12].

### Gene structure analysis:

To construct the genomic structure of the proposed genes, the online Gene Structure Display Server (GSDS 2.0, http://gsds.cbi.pku.edu.cn/index.php) was used [24]. The gene structure of the proposed CaDCL genes of C. arabica was constructed along with the AtDCL genes from *A. thaliana* to compare the structure. These combined gene structure figures exhibited the composition of exon-intron and numbers of intron were recorded from GSDS.

### Sub-cellular localization analysis:

The cellular location of the gene product was investigated into the cell organelles to improve the understanding about the functional mechanism of the DCL genes in C. arabica. In this purpose, an online integrative subcellular location predictor tool called plant subcellular localization integrative predictor (PSI) [25] was utilized to identify the location of the DCL genes of Coffee plant.

### Cis-regulatory element identification:

The cis-regulatory element associated with newly identified DCL genes were retrieved which are one of the key components regarding the transcription process in eukaryotes. The cis-regulatory elements were searched against the CaDCL into the PlantCARE database (http://bioinformatics.psb.ugent.be/ webtools/plantcare/html/), which is a database of plant promoters and cis-acting regulatory elements. The identified cis-acting element were classified and represented according to their activities like light responsiveness (LR), hormone responsiveness (HR), stress responsiveness (SR) and other responsiveness.

## Result and Discussion:

To identify the RNAi pathway genes in C. arabica, all the downloaded sequences were examinedto check their properties compared to the Arabidopsis DCLs.Finally5CaDCL genes were recognized in the coffee genome. The genomic information of the identified genes exhibited that the protein length of the CaDCLs varied from 1553 (CaDCL3b) to 2604(CaDCL4b). The number of intron of the CaDCL genes varied from 20 (CaDCL1) to 26 (CaDCL4b) (Table 1). TheCaDCL genes have maximum genome length 25581bp (CaDCL4b) while the minimum genome length is 10701bp (CaDCL1).

**Figure 2:**
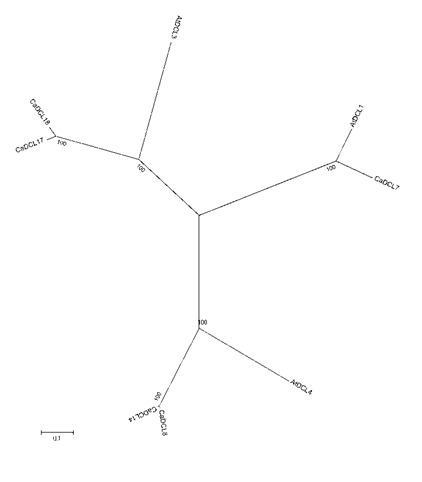
The evolutionary relationship analysis of the reported RNAi proteins of the Coffee. The phylogenetic relationship revealed three subgroups for CaDCL in coffee plant. The accession numbers of AtDCL proteins from Arabidopsis are given below: AtDCL1 (At1g01040), AtDCL2 (At3g03300), AtDCL3 (At3g43920) and AtDCL4 (At5g20320).

In order to conduct the phylogenetic relationship analysis between the DCL proteins of C. arabica and Arabidopsis, the total length amino acid sequences of these plants were used. The neighbor-joining method along with the 1000 bootstrap values was considered to phylogenetic tree. The phylogenetic analysis of the identified genes showed 3 clusters along with the DCL genes of A. thaliana. The CaDCL genes were named as CaDCL1, CaDCL3a CaDCL3b, CaDCL4a and CaDCL4b. In this study no DCL2 candidate was found in the coffee genome. The evolutionary relationship study revealed that the DCL3 and DCL4 have two more members, which are named as CaDCL3b and CaDCL4b accordingly (Figure 2).

**Figure 3:**
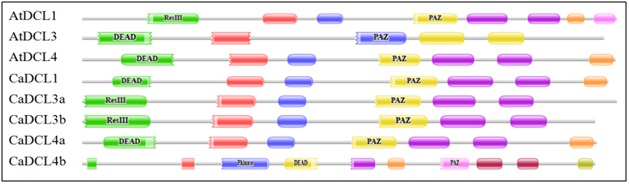
The conserved domain of the reported CaDCL genes in Coffee plant obtained from Pfam database (http://pfam.sanger.ac.uk/)

The conserved domains of the CaDCL genes were checked through the NCBI-CDD and Pfam database separately. The entire CaDCL genes showed the conserved domains like DEAD, Helicase_C, Dicer_dimar, PAZ, RNase III and DSRM which are the core functional domain part of DCL genes (Figure 3). All the CaDCL contained the Ribonuclease_3 domain which is the main domain part that participates into the cleavage activities to trigger the double stranded RNA into small short interfering RNA (Table 1)[26]. All the six types of conserved domains DEAD, Helicase_C, Dicer_dimar, PAZ, RNase III and DSRM were shared by all the CaDCl genes in Coffee plant. All the CaDCL genes exhibited two RNase III type domains, which have been considered one of the key domains for DCL genes [26,27]. Along with these domains, the CaDCL4b also shared LRRNT_2, LRR_8, P kinase domains which may play significant role in DCL activities in Coffee plant. These three type of domain were not found before in others DCL genes those were reported in previous studies.

**Figure 4:**
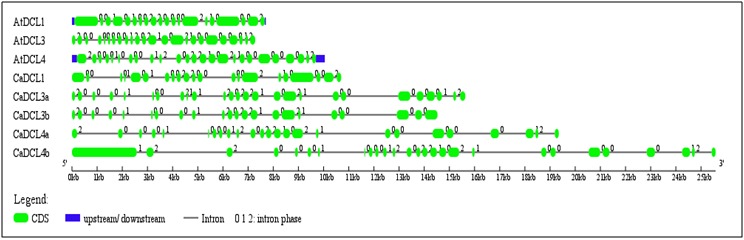
Gene structure of the CaDCL genes in *C. Arabica* retrieved using Gene Structure Display Server.

The gene structure of the CaDCLs gene was retrieved through the GSDS server to observe their exon-intron configuration. The gene structures of the corresponding AtDCL genes of Arabidopsis were also shown with the CaDCLs genes (Figure 4). It was observed that the exon-intron configuration of the reported genes were symbolized the similarity with the AtDCLs. The gene structure of CaDCLs exhibited 21-26 introns correspondence according to higher similarity with AtDCLs. The genomic location of the reported genes was scattered into five distinguish scaffold into the whole genome of the C. arabica.

**Figure 5:**
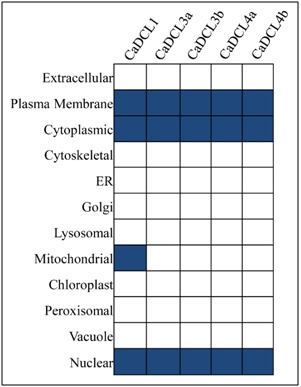
The sub-cellular localization analysis for the reported CaDCL proteins.The protein appeared in different cellular components is represented in deep coloured boxes

To decipher to the cellular appearance of the reported proteins were investigated through the sub cellular localization studies. The sub-cellular localization analysis showed that entire CaDCL proteins are distributed in nucleus, cytoplasm and plasma membrane (Figure 5). The transcriptional gene silencing (TGS) happens in nucleus as well as post transcriptional gene silencing (PTGS) occurs into the cytoplasmic region [28] of the cell. The RNA polymerase type II complexes are directly involved [29] in protein transcriptional procedure. The appearance of the CaDCL genes reflected that they are associated with the TGS and PTGS into the cell. The CaDCL1 also appeared into the mitochondria. The scatterings of the reported CaDCL into the cell nucleus and cytoplasm revealed that they have a direct involvement in the RNAi process in C. arabica.

**Figure 6:**
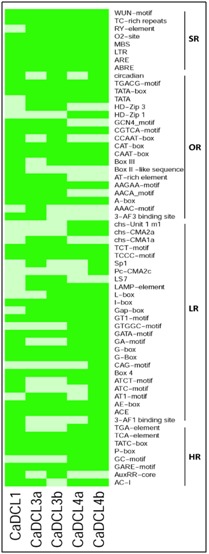
The cis-acting regulatory element associated with the reported CaDCL genes in Coffee plant. Here green rectangular represents the existing of corresponding cis-regulatory element with the associated CaDCL

The in silico identification of the cis-acting regulatory element associated with the CaDCLs were retrieved numerous informative motifs in *C. Arabica* through the Plant CARE database. The identified cis-acting elements were classified into five groups as light responsiveness (LR), hormone responsiveness (HR), stress responsiveness (SR), others responses and unknown functions according to their functionality into the cell. Among the detected known biological functions of the cis-elements, it was found that most of them were light responsive (LR) (Figure 6). The common light responsive motifs shared by the CaDCL genes in c. Arabica are ACE (cis-acting element involved in light responsiveness), AE-box (part of a module for light response), Box 4 (part of a conserved DNA module involved in light responsiveness), GATA-motif (part of a light responsive element), GT1-motif, I-box, TCCC-motif, TCT-motif (light responsive element). In Coffee plant genome the AC-I, AuxRR-core, GARE-motif, GC-motif, P-box, TATC-box, TCA-element and TGA-element motifs were identified as the hormone responsive cis-acting regulatory elements, which actually involved in plant phyto hormones processing (6). The involvement of the cis-elements of the reported CaDCL in light responsiveness and hormone responsiveness clearly indicated the close relationship with the plant photosynthesis and plant growth and development.

On the other hand, the ABRE, ARE, LTR (cis-acting element involved in low-temperature responsiveness), MBS (MYB binding site involved in drought-inducibility), O2-site, RY-element, TC-rich repeats (cis-acting element involved in defence and stress responsiveness) and WUN-motif (wound-responsive element) were found as the stress responsive cis-acting element of reported CaDCL (Figure 6). In addition, the cis-element are also displayed various significant motifs such as, part of a conserved DNA module array (CMA3) (3-AF3 binding site), sequence conserved in alpha-amylase promoters (AAAC-motif), protein binding site (Box III), common cis-acting element in promoter and enhancer regions (CAAT box), cis-acting regulatory element involved in the MeJA-responsiveness (TGACG-motif) and others (Figure 6). Some unknown motifs were found with their unknown biological function in C. arabica genome (Supplementary file 1 available athttp://www.bbcba.org/softwares/Coffee_DCL.zip). The cis-acting element analysis indicates that the proposed CaDCL genes may exhibit a diverse expression pattern, which can be observed by experimental analysis of the reported genes.

## Conclusion

The coffee is considered as one of the most important agricultural commodity worldwide [30]. The impact of world climate change affects the perennial cropping systems, which demand the genetic development of the plant in various adverse stress conditions using the coffee cultivation system [31-34]. We report five CaDCL proteins with relevant genetic data for further verification. The gene structure, functional domain composition, subcellular location as well as the genetic regulatory elements are described. The phylogenetic diversity analysis showed three sub classes of the DCL genes in C. arabica. The gene structure, functional domain was compared with the counterpart of Arabidopsis DCL genes, which revealed maximal homogeneity with the AtDCL. The sub cellular location showed that the CaDCL proteins are scattered throughout the cell. The cis-acting regulatory elements link with diverse biological functions in Coffee plants during plant growth and development in various stages in its life cycle. Data on CaDCL provides a basis for further analysis on RNAi pathway genes in Coffee plant to enrich plant development and Coffee production worldwide.

## Figures and Tables

**Table 1:** The basic genomic information about the reported CaDCL genes in Coffea arabica

Gene Name	Accession No.	Gene Location	Gene Length	No. of Intron	Protein Length	Protein Domains
			(bp)		(AA)	
CaDCL1	evm.model.Scaffold_548.2	Scaffold_548:177015..187715	10701	20	1737	DEAD, Helicase_C, Dicer_dimer, PAZ, Ribonuclease_3, Ribonuclease_3, DND1_DSRM
CaDCL3a	evm.model.Scaffold_1082.215	Scaffold_1082:3503760..3519391	15632	25	1709	ResIII, Helicase_C, Dicer_dimer, PAZ, Ribonuclease_3, Ribonuclease_3
CaDCL3b	evm.model.Scaffold_2631.86	Scaffold_2631:1936073..1950599	14527	21	1553	ResIII, Helicase_C, Dicer_dimer, PAZ, Ribonuclease_3, Ribonuclease_3
CaDCL4a	evm.model.Scaffold_571.818	Scaffold_571:5929371..5948718	19348	24	1643	DEAD, Helicase_C, Dicer_dimer, PAZ, Ribonuclease_3, Ribonuclease_3, DND1_DSRM
CaDCL4b	evm.model.Scaffold_671.1174	Scaffold_671:9594104..9619684	25581	26	2604	LRRNT_2, LRR_8, Pkinase, DEAD, Helicase_C, Dicer_dimer, PAZ, Ribonuclease_3, Ribonuclease_3
